# Solitary fibrous tumor of the spermatic cord: A case report and literature review

**DOI:** 10.3892/etm.2014.2066

**Published:** 2014-11-12

**Authors:** SHANBIAO HU, LU YI, LUOYAN YANG, YINHUAI WANG

**Affiliations:** Department of Urology, Second Xiangya Hospital, Central South University, Changsha, Hunan 410011, P.R. China

**Keywords:** mesenchymal tumor, solitary fibrous tumor, spermatic cord

## Abstract

A 31-year-old male patient with a six-year history of left inguinoscrotal swelling was admitted to the Second Xiangya Hospital (Changsha, China). The mass was not found to be associated with intraperitoneal pressure. Ultrasonography and computed tomography examinations demonstrated several solid, botryoidal masses involving the spermatic cord, with limited capacity of mobility. The demarcation between the masses and the left testicle was clear; thus, the masses were removed by a left spermatic cord tumor resection via a left inguinal approach, under epidural anesthesia. Positive staining of the tumor markers, CD34^+^, CD99^+^ and Bcl-2^+^, was confirmed by pathological examination following surgery, and a solitary fibrous tumor of the spermatic cord was diagnosed. No recurrence and metastasis were observed in the patient during the subsequent 25-month follow-up period.

## Introduction

Solitary fibrous tumors (SFTs) are a rare type of mesenchymal tumor, which were first described by Klemperer and Rabin in 1931 (1). Although they are commonly found in the pleura, statistics show that 50–70% of all SFTs are extrapleural (2). However, few cases of SFTs in the urogenital organs have been documented. To the best of our knowledge, only a small number of these cases forming in the spermatic cord have been reported to date (3–7).

Due to the rarity of these tumors, there was no consensus on its standard treatment. However, treatment for SFTs include surgical resection, radiotherapy and chemotherapy. Resection of the tumor with a sufficient surgical margin was the mainstay of treatment. However, a number of studies have demonstrated that radiotherapy and chemotherapy, with ifosfamide and adriamycin, is effective (5,8). However, these treatments were not established yet. The present study reports a case of SFT involving the spermatic cord.

## Case report

The patient was a 31-year-old male with a six-year history of left inguinoscrotal swelling, which had been gradually increasing in size for the past three years. The size of the swelling showed no association with increased intraperitoneal pressure. Physical examination revealed no evident defects in the appearance of the scrotum, with the exception of several hard, botryoidal masses, located between the left testicle and the inguinal region, with smooth surfaces and limited capacity of mobility. Ultrasound examination revealed several solid, well-demarcated, hypoechoic extratesticular masses arising from the left spermatic cord, the largest of which was ~3 × 2 cm in size. The masses were separated from the cutis and showed no connections with the left testis and epididymis, and no vascularity was observed. The patient’s family signed an informed consent prior to the treatment.

**Figure 1:**
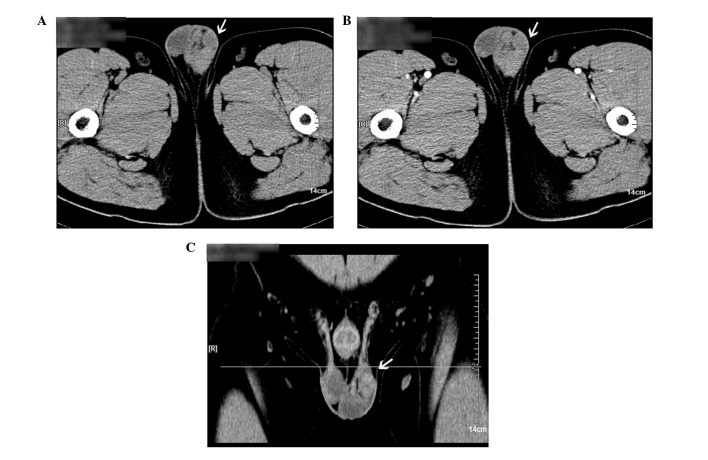
CT images of an SFT in the spermatic cord: (A) Unenhanced CT scan; (B) enhanced CT scan (the mass density is enhanced slightly); and (C) coronal section of the enhanced CT scan. SFT, solitary fibrous tumor; CT, computed tomography.

A computed tomography (CT) scan revealed multiple high-density nodules associated with the left spermatic cord, which showed partial fusion, uniform density and a clear boundary with the testis (Fig. 1). In addition, the density of the nodules was enhanced slightly and no bilateral inguinal swollen lymph nodes were observed in the enhanced scan.

**Figure 2:**
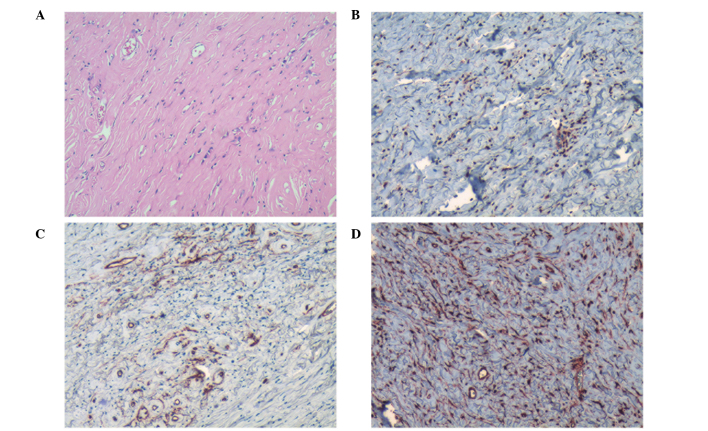
Immunohistochemical detection of tumor marker expression, showing (A) hematoxylin-eosin staining, (B) Bcl-2^+^, (C) partial CD34^+^ and (D) CD99^+^. Magnification, ×100.

The masses were removed by a left spermatic cord tumor resection via a left inguinal approach, under epidural anesthesia. Benign mesenchymal tissue was diagnosed by intraoperative frozen pathological sections. Postoperative pathological examination confirmed that the masses belonged to multiple mesenchymal tumors with classic properties, including fibroplasia, small vascular hyalinization, clear boundaries, less tumor component and no nuclear division. Immunohistochemical examination revealed the presence of numerous tumor markers, including CD99^+^, Bcl-2^+^, partial CD34^+^, focal S-100^+^, SMA^+^ and CD68^−^ (Fig. 2). Thus, the final diagnosis was solitary fibrous tumors of the spermatic cord. The patient remained healthy, with no local recurrence or metastasis observed during the 25-month follow-up period.

## Discussion

Although SFTs have been observed in male and female patients of a wide range of ages, the underlying mechanism remains unknown. Initially, SFTs were most commonly located in the pleura (9). At present, only a limited number of SFT cases have been identified in the urogenital organs, including the kidney, renal pelvis, bladder, prostate and seminal vesicles (4,6,7,9–12). Moreover, SFTs involving the spermatic cord are extremely rare and should be distinguished from other urogenital mesenchymal tumors, which include inflammatory myofibromatous tumors, hemangiopericytomas, malignant fibrohistiocytomas, fibrosarcomas, leiomyosarcomas and benign and malignant nerve sheath tumors. In addition, SFTs should be distinguished from other diseases that can result from inguinal masses, similarly to an inguinal hernia (11).

Clinical symptoms of urogenital SFTs, including a painless mass, dysuria, hematospermia and abdominal pain, are mainly determined by tumor properties, including the size, location, invasion and the degree of mucosal integrity (11).

Ultrasonography and magnetic resonance imaging (MRI), two common imaging examinations, play an important role in the diagnosis of SFTs. Ultrasound examination is more effective in the identification of solid and cystic tumors, while MRI is able to illustrate anatomical associations in the tumor surroundings more precisely (7). Isodensity with patchy hypodensity for unenhanced CT images, and mild or marked heterogeneous enhancement for contrast-enhanced CT images are the major features of pleural SFTs (13,14). A study by Zhang *et al* demonstrated that a well-defined, ovoid or rounded mass with low signal intensity on MRI T2-weighted images and different degrees of enhancement on CT and MRI scans may indicate the presence of an SFT (14). Positron emission tomography-CT scanning is able to preoperatively indicate malignancy and recognize local recurrence or distant metastases; however, Lococo *et al* deemed that it is controversial for the identification of benign and malignant SFTs for PETCT. Thus, further evaluation is required (15,16). In the present study, an unenhanced CT scan demonstrated multiple high-density nodules, and the signal only strengthened slightly in the enhanced CT scan. Therefore, the diagnosis of an SFT by imaging only is inadvisable and further pathological examinations are necessary.

Previous studies have confirmed that tumor markers, including CD34^+^, Bcl-2^+^ and CD99^+^, are generally positive in SFT cases, which has aided the diagnosis of SFTs (17,18). Currently, the positive staining of CD34^+^ is indispensable in the diagnosis of an SFT; however, the expression of CD34 is not limited to SFTs (11). In the present study, the positive staining of CD34^+^, Bcl-2^+^ and CD99^+^ was demonstrated by immunohistochemistry examinations, confirming the diagnosis of an SFT.

The conventional treatment for an SFT is the complete resection of the tumor with negative margins (19,20) or resection experience satisfactory health outcomes (21). However, the resection of tumors with positive margins is associated with a higher rate of local recurrence, and surgical excision remains the first choice for the treatment of local recurrence (20). With regard to urogenital SFTs, the selection of the surgical procedure should depend on the characteristics of each individual tumor, including the location, size and degree of malignancy. For tumors with infiltrative growth, of a large size or aggressive malignancy, the recommended treatment is the extended resection of the involved organs, including radical nephrectomy and cystoprostatectomy. In the present study, considering the clear demarcations between the tumors and surrounding organs, complete resection of the tumors was performed without removing the spermatic cord.

The prognosis of SFTs is obscure for rare cases. Usually, an SFT is defined as a benign form of tumor due to its low rates or even absence of local recurrence and metastasis, as demonstrated by a number of previous studies (1,2,22,23). However, an increasing number of recent studies have demonstrated that a considerable number of SFTs are malignant (11,18,24). In the case of an SFT which is unresectable, metastatic or has positive surgical margins, patients are unlikely to benefit significantly from radiotherapy or chemotherapy; the alternative treatments for an SFT (2). Therefore, a careful long-term follow-up period is advised in all SFT cases due to the possibility of a late recurrence (21). The case reported in the present study was considered to be a benign SFT due to its small size (<10 cm), lack of growth and minimal cancer component. During the 25-month follow-up period, the patient remained healthy and exhibited no tumor recurrence.

In conclusion, pathological examination remains the standard approach for the diagnosis of SFTs, while radical resection of the tumor/s is the first choice for treatment. Furthermore, patients should undergo careful follow-up examination in order to identify tumor recurrence.
